# Comparison of silicone versus polyurethane ureteral stents: a prospective controlled study

**DOI:** 10.1186/s12894-020-0577-y

**Published:** 2020-02-03

**Authors:** Nariman Gadzhiev, Dmitry Gorelov, Vigen Malkhasyan, Gagik Akopyan, Revaz Harchelava, Denis Mazurenko, Christina Kosmala, Zhamshid Okhunov, Sergei Petrov

**Affiliations:** 1 Department of Urology, Pavlov First Saint Petersburg State Medical University, Saint Petersburg, Russia; 2 Department of Urology, State University of Medicine and Dentistry, Moscow, Russia; 3 Institute for Urology and Reproductive Health, Sechenov University, Moscow, Russia; 4 Department of Urology, Ilyinskaya Hospital, Moscow, Russia; 5 Department of Urology, University of California, Irvine, 333 City Boulevard West, Orange, CA 92868 USA

**Keywords:** Ureteral stent, Silicone, Polyurethane, Stent-related symptoms, Quality of life

## Abstract

**Background:**

Approximately 80% of patients with indwelling ureteral stents experience stent related symptoms (SRS). We believe SRS can be reduced through altering the composition of ureteral stents to a less firm material. Therefore, we aim to compare modern silicone and polyurethane ureteral stents in terms of SRS intensity and safety.

**Methods:**

From June 2018 to October 2018, patients from two distinct clinical centers were prospectively enrolled in the study and stratified (non-randomly) into either control group A, patients who received polyurethane stents (Rüsch, Teleflex), or experimental group B, patients who received silicone stents (Cook Medical). Each participant completed a survey 1 h after stent insertion, in the middle of the stent dwelling period, and before stent removal or ureteroscopy noting body pain and overactive bladder via the visual analog scale pain (VASP) and overactive bladder (OAB) awareness tool, respectively. Additionally, successfulness of stent placement, hematuria, number of unplanned visits, and stent encrustation rates were assessed within each group.

**Results:**

A total of 50 patients participated in the study, control group A consisted of 20 patients and experimental group B consisted of 30 patients. Participants in group B, silicone ureteral stents, demonstrated significantly lower mean values of VASP 2 weeks prior to stent removal and promptly before stent removal (*p* = 0.023 and *p* = 0.014, respectively). No other comparisons between the two groups were statistically significant.

**Conclusions:**

Compared to polyurethane ureteral stents, silicone ureteral stents are associated with lower body pain intensity assessed by VASP 2 weeks before stent removal and at the time of stent removal.

**Trial registration:**

Current Controlled Trials NCT04000178. Retrospectively registered on June 26, 2019.

## Background

Double-J ureteral stents have become a fundamental endourological treatment since Roy P. Finney introduced them in 1978 [1]. In the United States, approximately 92,000 ureteral stents are placed annually to manage upper urinary tract obstructions caused by urolithiasis and other genitourinary pathologies [2]. While most indwelling stents aid patients with drainage, over 80% of patients with indwelling stents experience stent related symptoms (SRS), such as storage symptoms and pain, which lower their quality of life (QoL) [3, 4]. Several studies have been conducted comparing different variations of stents characteristics and the impact each had on patients experiencing SRS. Through these studies, physicians can better individualize ureteral stents for each patient in order to decrease SRS. These characteristics include the length of the stent [5], stent positioning [6], changing bladder pig tail to loop type [7], use of stents with special coatings [8], and composition of biomaterials of different firmness [9]. Additionally, the use of alpha1- and choline blockers, with their combinations [10], nonsteroidal antiinflammatory drugs [11] and mirabegron [12] have also been studied in order to reduce intensity of SRS experienced by patients. Despite this, patients with indwelling stents continue to experience SRS [13].

In a study by Lennon et al., a significant increase in SRS was attributed to ureteral stent firmness [12]. The magnitude of stent firmness is reliant on the material ureteral stents are composed of. Today, ureteral stents are primarily composed of polyurethane due to a low fracture propensity and high tensile strength [14]. However, when ureteral stents were first introduced in 1978, they were composed of silicone [15]. Compared to modern polyurethane ureteral stents, silicone ureteral stents were softer, more biocompatible [16], and have lower encrustation rates [17]. Despite these benefits, silicone ureteral stents were ultimately replaced with polyurethane ureteral stents due to higher frictional forces during placement [15] and smaller side holes as a result of lower tensile strength. However, technological advancements have made it possible to produce silicone ureteral stents similar to polyurethane ureteral stents. With these advancements we believe that adopting modern silicone ureteral stents could reduce SRS and as a result increase patient’s quality of life (QOL) with indwelling ureteral stents. This study aims to compare patient discomfort between modern silicone and polyurethane ureteral stents.

## Material and methods

After institutional review board approval (IRB), informed consent was obtained from 50 patients admitted with acute renal colic from June 2018 till October 2018 at two academic institutions. Inclusion criteria included age (18 to 60 years old), confirmed ureteral stone and prescribed ureteral stent placement for pain syndrome relief. Patients were excluded from the study if they had an active urinary tract infection. Participating patients were then divided into two groups: group A (*n* = 20) – patients who received polyurethane stents (Rüsch, Teleflex) – and group B (*n* = 30) – patients who received silicone stents (Cook Medical). All patients received 6 Fr, 26 cm ureteral stents and were placed via cystoscopy and X-ray guided control under total intravenous anesthesia with propofol and fentanil. Stent indwelling time lasted for 4 weeks during which each patient underwent a follow-up assessment 1 h after insertion, in the middle of the stent dwelling period, and before ureteroscopy or stent removal.

### Outcome measurement

The primary outcome of this study was the assessment of body pain and bladder irritation. Secondary outcomes of the study were success of stent placement, hematuria, unplanned visits and stent encrustation, defined by urologist at the time of the stent removal. At each follow-up appointment, patients completed a survey in which the visual analog scale pain (VASP) and overactive bladder (OAB) awareness tools were used to assess the intensity of SRS. Both of these tools have been previously validated for body pain and overactive bladder symptoms assessment [18, 19].

The ureteral stent symptom questionnaire (USSQ), which is considered as a gold standard for evaluating SRS, was not used since there is no validated Russian version.

### Statistical analysis

For all parameters, 95% confidence intervals (CIs) were calculated. То calculate CIs for the differences and/or ratio, the MOVER approach was used implemented in the spreadsheets: MOVER-D.xls and MOVER-R.xls (http://profrobertnewcomberesources.yolasite.com/). To present interval estimations the compact format was used in which the lower and upper limits are shown as subscripts surrounding the point estimate [20]. PAST software was used to test the agreement of the observed values with the normal distribution and for the statistical estimation of the parameters and their comparisons [21]. For the analysis of discrete data the exact nonparametric methods were used implemented in StatXact package (http://www.cytel.com/software/statxact). Boxplots with whiskers and notches were drawn using online BoxPlotR (http://shiny.chemgrid.org/boxplotr/). A *p*-value of < 0.05 was considered statistically significant.

## Results

A total of 50 patients underwent stent insertion and were stratified as follows: 20 pts. received polyurethane stents and 30 pts. silicone stents. Their demographic and clinical data are presented in Table 1. Both groups appeared to be statistically homogeneous except the stone size, which was statistically larger in Polyurethane group (*p* = 0.001), and we didn’t regard it as relevant to the aim of the study.

**Table 1:** Demographic and clinical data

	Polyurethane	Silicone	*p*-value
Patients, n (%)	20 (40%)	30 (60%)	
Age (years); median (range)	50 (19–60)	48 (24–64)	0.96
Male, n (%)	7 (35%)	18 (60%)	0.19
Female, n (%)	13 (65%)	12 (40%)	
BMI (kg/m^2^)	_26_ 26 _31_	_26_ 28 _30_	0.71
Stone size (S) (mm)	_9_ 12 _13_	_6_ 8 _10_	**0.001**
Stent installation time, min	_6.1_ 7.8 _9.4_	_6.8_ 7.8 _8.9_	1.00
Duration of stay of the stent, weeks	_3.2_ 3.5 _3.7_	_3.6_ 3.8 _4.0_	0.015

*p*-values in the last column are for the comparisons between groups

Comparison of VASP and OAB awareness tool at 1 h after stent insertion, in the middle of the stent dwelling period and before ureteroscopy or stent removal **(**Table 2**)** demonstrated significant differences between mean values of VASP at 2 weeks and before stent removal in favor of group B (silicone ureteral stents) (*p* = 0.023 and *p* = 0.014, respectively).

**Table 2:** Comparison of VAS Pain, OAB awareness tool and EQ-5D-5 L questionnaire at three points: 1 h and 2 weeks after insertion, and before stent removal

VASP, scores			
1 h	_1.4_ 2.8 _4.0._	_1.4_ 2.0 _2.5_	0.23
2 weeks	_1.3_ 2.4 _3.4_	_0.7_ 1.1 _1.5_	**0.023**
Before	_1.3_ 2.1 _2.9_	_0.8_ 1.1 _1.4_	**0.014**
*p*-value	0.25	**0.0029**	
OAB, scores			
1 h	_4.2_ 7.2 _10_	_4.7_ 6.3 _7.8_	0.61
2 weeks	_5.1_ 8.2 _11_	_4.8_ 6.2 _7.2_	0.25
Before	_5.7_ 8.7 _12_	_5.0_ 6.8 _8.5_	0.27
*p*-value	0.48	0.76	

Data are shown as medians; the lower and upper limits of 95%CI are given as left and right subscripts. *p*-values in the last column are for the comparisons between groups; *p*-values in rows are from ANOVA for the repeated measurements at three-time points. Statistically significant values are in bold

In order to specify the difference between the groups score totals were calculated and compared **(**Table 3**).** Mean VASP scores in Silicone group were significantly lower than in Polyurethane group (*p* = 0.0010). It should be noted that polyurethane stents lead to an abnormally high variance in scores in comparison to silicone stents. This means that the results with the polyurethane stents appeared to be more variable, uncertain and unpredictable than with the silicone stents.

**Table 3:**
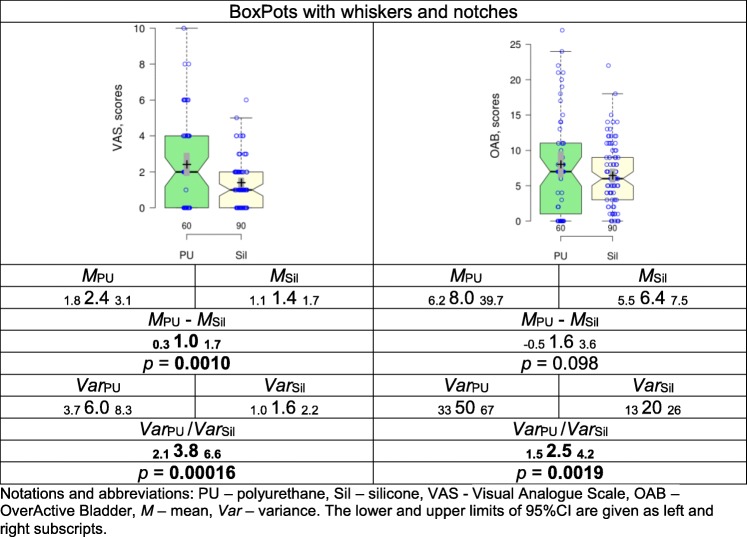
Comparison of the score totals of VASP and OAB in the patients with the polyurethane and silicone stents

Notations and abbreviations: *PU* polyurethane, *Sil* silicone, *VAS* Visual Analogue Scale, *OAB* OverActive Bladder, *M* mean, *Var* variance. The lower and upper limits of 95%CI are given as left and right subscripts

No statistically significant difference was observed between groups comparing secondary outcomes **(**Table 4**).**

**Table 4:** Secondary outcomes comparison between groups

Secondary outcome	Stent		*p*-value
	Polyurethane	Silicone	
Difficult stent placement			
No	13 (64%)	26 (85%)	0.09
Yes	7 (36%)	4 (15%)	
Unplanned visits			
No	17 (83%)	30 (100%)	0.052
Yes	3 (17%)	0 (0%)	
Encrustation			
No	17 (83%)	29 (95%)	0.29
Yes	3 (17%)	1 (5%)	
Hematuria			
No	9 (45%)	19 (63%)	0.25
Yes	11 (55%)	11 (37%)	

## Discussion

In 1967, Dr. Paul Zimskind first reported using ureteral silicone tubing as an indwelling stent. In theory, these stents were to provide aid with drainage for up to 19 months however, some were expelled due to the absence of a mechanism to prevent stent migration [22]. Seven years later, Gibbons presented a silicone stent equipped with a distal flange and sharply pointed barbs to prevent upward and downward migration, respectively. Nonetheless, this design was difficult to insert into patients because the barbs increased the stent size from 7 Ch to 11 Ch [2]. Then in 1978, Roy P. Finney presented the double pigtail silicone stent design that most resembles the ureteral stents used in practice today.

Ureteral stents are mainly used to relieve urinary obstruction and are used in a variety of cases such as obstructing stones, strictures, and aid in effective drainage [23]. Despite their benefits, over 80% of patients with indwelling ureteral stents experience stent-related pain that affects their ability to perform daily activities, causing at least one-third of patients to prematurely remove their ureteral stents [24]. Premature removal of ureteral stents can alternatively affect patient recovery and lead to more post-operative complications. Thus, to improve surgical outcomes and patient health, it is important to limit SRS. In a study conducted by Lennon et al. [25], it was concluded that the softness of the ureteral stent directly influenced patients’ tolerability; stents made from softer material correlated with less incidence of dysuria and pain [15]. Additionally, in a recent study found that silicone ureteral stents were associated with less patient discomfort at day 20 Post OP [26]. Therefore, in order to further reduce SRS, alterations to stent composition could be a solution.

In this study, the intensity of SRS in traditional polyurethane ureteral stents and modern silicone ureteral stents were compared. From the data, use of the OAB awareness tool proved unfit to accurately assess patient QoL. However, VASP data concluded that patients who received silicone ureteral stents had a significantly better QoL in the middle of the stent indwelling period and immediately prior to stent removal compared to those who received polyurethane ureteral stents (*p* = 0.023 and *p* = 0.014, respectively).

Despite these findings, some studies have not found a correlation between stent material composition and patient QoL [27–29]. Due to these inconsistent findings, additional long-term research studies should be completed before any conclusive statements can be made regarding stent material and the affect it has on QoL. Nonetheless, none of these studies specifically compared silicone ureteral stents to polyurethane stents. This could be mainly due to recent advancements in modern technology that have made it possible to manufacture silicone ureteral stents with the same external diameter, internal diameter, and size of side holes as in polyurethane ureteral stents. Due to these advancements our study displayed the superiority of silicone ureteral stents in terms of body pain at 2 weeks before stent removal and immediately before stent removal.

Comparing stent-related complications from both Group A and Group B, no significant difference was observed. These results speak to the overall safety of silicone ureteral stents. Originally, silicone ureteral stents were replaced with polyurethane ureteral stents due to low tensile strength which limited the internal diameter and aperture of side holes. Additionally, silicone ureteral stents proved more difficult to place due to a high volume of friction and were more expensive to produce leading to the switch to polyurethane ureteral stents [30]. Because of recent technological advancements, silicone stents are beginning to become more similar to traditional polyurethane ureteral stents in both size and safety. In our study there was no significant difference in the stent encrustation rate. This could have been due to the small sample size used and short duration of the study limited to 4 weeks. A larger sample size and longer study duration should be used to accurately determine if stent encrustation would differ between polyurethane and silicone ureteral stents.

Thus, silicone ureteral stents may be a viable option for patients who have had a previous negative experience with polyurethane ureteral stents or could alternatively be used as a replacement stent for patients with current indwelling polyurethane ureteral stents experiencing SRS. However, silicone ureteral stents depending on country can be more expensive than polyurethane ureteral stents as in Russia, which can be an obstacle receiving such stents.

Our study did have some limitations: it was a non-randomized study with a small sample size which may have affected our study results. Additionally, groups differed in terms of stone size which could possibly affect successfulness of stent placement. One of the instruments for assessment of SRS was OAB awareness tool, which is not very popular but has Russian validated translation. Further research is needed to confirm the results of our study.

## Conclusions

Silicone ureteral stents were associated with lower body pain intensity assessed by VASP 2 weeks prior to stent removal and immediately prior to stent removal in comparison with polyurethane ureteral stents.

## Data Availability

The datasets used and/or analyzed during the current study are available from the corresponding author on reasonable request.

## Abbreviations

OAB: Overactive bladder
PU: Polyurethane
QoL: Quality of life
SRS: Stent related symptoms
VASP: Visual analog scale pain
